# COVID-19 Vaccine Hesitancy Among Healthcare Workers: A Phenomenological Study of Skepticism

**DOI:** 10.7759/cureus.58445

**Published:** 2024-04-17

**Authors:** Parvathy Thampy, Shweta Sharma, Pragya Joshi, Munna S Raj, Ashlesh Rupani, Shivank Tyagi, Ankur Joshi

**Affiliations:** 1 Community and Family Medicine, All India Institute of Medical Sciences, Bhopal, Bhopal, IND; 2 Community Medicine, Lakshmi Narain Medical College and Research Centre, Bhopal, IND; 3 Department of Training & Placement, Oriental Institute of Science and Technology, Bhopal, IND; 4 Internal Medicine, KIMSHEALTH, Thiruvananthapuram, IND; 5 Community Medicine, Government Medical College Jalgaon, Jalgaon, IND; 6 Rajbhasha Department, All India Institute of Medical Sciences, Bhopal, Bhopal, IND

**Keywords:** text mining, vaccine psychology, health personnel, covid-19 vaccine, vaccine hesitancy

## Abstract

Rationale: Despite the prioritizing the healthcare workers (HCWs) for COVID-19 in a systematized manner the phenomenon of vaccine hesitancy was observed in them. HCWs are presumed to be pre-emptive in up-taking the vaccine due to their closest association and having reasonable background information. Hence, we intended to explore and investigate the phenomenology of skepticism and hesitancy toward the COVID-19 vaccine among HCWs.

Method: A sequential explanatory mixed methods study design incorporating a baseline cross-sectional survey followed by qualitative and semiquantitative text-mining approach was adopted in a tertiary care center in Madhya Pradesh, India. Six hundred seventy-nine HCWs for quantitative data and 30 HCWs for qualitative interviews were surveyed. After determining the quantum and baseline traits of hesitant HCWs, 30 participants were purposively selected for in-depth qualitative analysis based on grounded theory using a framework approach and consolidated from the psychological and philosophical plane of skepticism. This was complemented by a semiquantitative text-mining approach using mono/bigram analysis and network plotting.

Results: Approximately one-fifth of participants (18%,122 out of 679) were initially, and one-tenth of initially hesitant (10 out of 122) were terminally hesitant. Hesitant and non-hesitant participants were similar except for comorbidity status. Five themes emerged namely individual, vaccine-related, social, system, and contextual after thematic consolidation. Words/phrases indicating individualistic desire to knowing more, internal conflicts, and conjecture were mined further. The network plot showed diversified expressions of participants.

Conclusion: There seems to be a requirement to prime HCWs by offering objective information beforehand and removing diffidence using a systematic approach addressing the psychology and prevalent partisan belief in similar circumstances in the future.

## Introduction

Coronavirus disease (COVID-19) has profoundly affected various aspects of life, including public health, the economy, and social well-being both globally and in India [1]. With its vast population, India has been significantly impacted by the COVID-19 pandemic both in terms of morbidity and mortality [2]. In the face of this overwhelming burden, healthcare workers (HCWs) have been at the forefront of providing essential preventive, promotive, and curative care.

Due to the nature of the profession, HCWs face a heightened risk of exposure to the SARS-CoV-2 [3]. Their close proximity and interaction to infected patients and households put them at increased susceptibility to COVID-19 infection. Consequently, HCWs are seven to 10 times more likely to develop severe COVID-19 infections compared to non-healthcare professionals [4,5]. This vulnerability may compromise the overall functionality and capacity of the healthcare system. Thus, it seems rational to prioritize HCWs for preventive vaccination against COVID-19. Vaccination is a proven strategy to reduce the risk of infection and to relenting severe illness [6]. However, it was observed that there is reluctance and hesitancy in this group as identified by several studies [7,8]. Further vaccine hesitancy was perceived as a significant barrier in achieving optimal vaccination coverage [9]. Thus, there seems to be a requirement to explore further the internal conflicts, fear, anxiety, doubt, and insecurity ingrained in these individuals.

With this study, we intended to explore the magnitude, distribution as well as phenomenology of COVID-19 vaccine hesitancy among HCWs of a tertiary care center in Madhya Pradesh, India using a pluralistic methodical approach. The findings from this study may be utilized for designing targeted interventions and strategies to address these barriers in similar circumstances.

## Materials and methods

This study was conducted among HCWs of tertiary care, dedicated COVID-19 hospital in Bhopal, Madhya Pradesh between October 2021 and August 2022.

**Figure 1:**
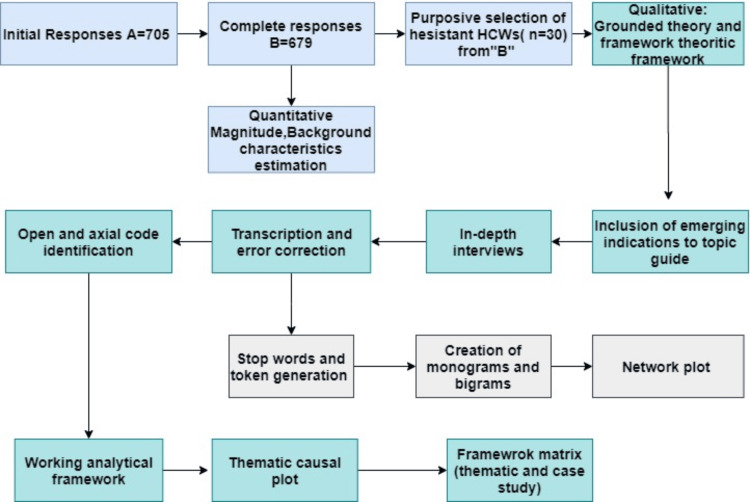
Process diagram showing methodology adopted

This study adopted a mixed methods sequential explanatory design. The research began with a quantitative survey conducted among HCWs addressing the magnitude of phenomenon and background characteristics of HCWs. The identified hesitant workers were selected for in-depth interviews to explore it further. For this study, we considered those HCWs as hesitant who did not receive their vaccination after 16 January 2021 (the launching of the nationwide drive) as per the slot offered to them by the designated vaccination center and in the absence of any contraindication. We chose the universal sample and purposive sample for the quantitative and qualitative part of the study respectively (Figure 1).

The survey tool consisted of sociodemographic, personal, occupational details of the participants. Information on assigned vaccination slot, type, frequency, and number of dosages were collected. The tool was validated for its content by three experts and was then pilot tested among 20 HCWs (Cronbach’s alpha=0.86). To ensure inclusivity and maximize participation, we administered this survey both in user-friendly web-based electronic form (self-filling modality) as well as an offline paper-based (assisted modality) format. An interview guide was developed after a thorough literature review in order to capture the phenomenon of vaccine hesitancy among HCWs, which consisted of questions as well as a set of probes wherever required. The same was translated into the Hindi language.

The phenomenon under inquiry (vaccine proclivity or hesitancy) took place in the real world and was emerging and evaluative in nature. Moreover, researchers wanted to capture the experience of hesitant people under the effect of this phenomenon (hesitancy) and then generate a theory of how the process works. This theory was supposed to be generated from data and the approach was nearer to the grounded theory approach. This approach offers a freeness from epistemological prejudices and is more inductive in nature. The topic guide consisted of four types of questions namely circumstantial, indicative, assessing, and premediated in nature. Data were collected through in-depth interviews. The questions (and probes) addressed the experiences, perceptions, and notions around hesitancy of workers. We adopted an iterative approach for the preparation of the topic guide with the inclusion of new questions in the light of emergence of new indications. The quantitative survey data was transformed into categories after checking for missing values, outliers, and redundancies. It was summarized through frequency distribution among hesitant and non-hesitant workers for baseline characteristics. A univariate analysis using non-parametric test was performed to detect any significant difference in baseline variables among the hesitant and non-hesitant HCWs.

Among the identified COVID-19 vaccine-hesitant HCWs, we selected 30 participants for in-depth interviews. We purposively selected all those 10 HCWs who opted out altogether of the vaccine and did not receive it at later days as well while the rest of the 20 participants had shown the initial vaccine hesitancy and had chosen to take the vaccine at later days. We chose HCWs across all strata of the cadre.

The data were transcribed using the Google Voice-to-Text Conversion facility and typographical errors were corrected manually. Analytical notes were prepared independently by two investigators, the data condensation was achieved by pasting the relevant piece of data by “open coding”. In the next step “axial codes” were constructed and further converged into the categories around what characterizes the vaccine hesitancy and the dimensions influencing the vaccine hesitancy. This prototype created the initial working analytical framework for indexing other transcripts. We honed this analytical framework cyclically during the data collection process. The data were condensed further by creating a matrix and charting the pieces of data in the matrix. A meaningful piece of information was abstracted by creating a matrix and allocating pieces of data to the matrix. The summarized description of the data was done as per theme (columns) and as per case (row). The semiquantitative text-mining process was started with relocating meaningful pieces of strings into a single word (tokenization). The noise of the data was removed by using stop-words. The team of investigators then identified the joint meaning of lost words through compound word identification. This was followed by the creation of mono and bigrams that provided the frequencies of connecting sequence of words for visualization through a bar diagram. The analysis showed a visual depiction of the frequentist method and contextual expressions. Then a network plot was created to identify patterns, trends, and associations between words and phrases. In the network diagram, each node represented an individual word or term extracted from textual sources. The edges between nodes illustrated correlations or co-occurrences between words and the thickness or darkness of the edges indicated the strength of the correlation.

The validity and reliability of the study were ensured by incorporating investigators from different backgrounds (public health and linguistics), universal inclusion of participants during the survey phase, and purposive selection of different health cadres having different education backgrounds and different work settings. The text data were connected with a coordinated comparison sheet and a respondent validation was done for selected verbatim. Analysis was done on R-environment (R version 4.3.1 (2023-06-16) and associated packages available freely in the public domain.

The study was initiated after getting approval from the Institutional Human Ethics Committee of All India Institute of Medical Sciences (AIIMS) Bhopal (IHEC-PGR/2021/PG/Jan/07).

## Results

Out of the 705 responses received during the survey, after excluding 26 missing responses, 679 HCWs data were included for analysis. The mean age of the participants was 28 years(SD=6.36 years, range=19-59 years), and 50.1% (n=340) were female. Most participants received Covishield (87%) followed by Covaxin vaccine (11.5%).

Among 679 HCWs, 122 HCWs (18%) did not take vaccine at their assigned slot, and 10 of them opted out of vaccine in later days as well. Hesitant HCWs were similar to their counterparts in sociodemographic, personal, and anthropometric characteristics except for reported comorbidities. The distribution of background characteristics in reference to hesitancy is depicted in Table 1.

**Table 1:** Background characteristics of participants in vaccine hesitant and vaccine non-hesitant group *Pearson's Chi-squared test ^#^Fischer Exact

		Initial Hesitation to COVID-19 Vaccine		
Characteristics	N(%) (N=679)	No (%) N=557	Yes (%) N=122	p-value
Age Category				0.349*
18-29	450 (66.3)	373 (67.0)	77 (63.1)	
30-39	171 (25.2)	137 (24.6)	34 (27.9)	
40-49	49 (7.2)	38 (6.8)	11(9.0)	
>50	9 (1.3)	9 (1.6)	0 (0.0)	
Gender				0.561^*^
Female	340 (50.1)	276 (49.6)	64 (52.5)	
Male	339 (49.9)	281 (50.4)	58 (47.5)	
BMI category				0.164^*^
Normal	456 (67.1)	366 (65.7)	90 (73.8)	
Obese	21 (31)	20 (3.6)	1 (0.8)	
Overweight	156 (22.9)	134 (24.1)	22 (18.0)	
Underweight	46 (6.8)	37 (6.6)	9 (7.4)	
History of smoking				0.997^*^
No	640 (94.3)	525 (94.3)	115 (94.3)	
Yes	39 (5.7)	32 (5.7)	7 (5.7)	
History of alcohol intake				0.531^*^
No	619 (91.2)	506 (90.8)	113 (92.6)	
Yes	60 (8.8)	51 (9.2)	9 (7.4)	
Comorbidities				0.001^#^
No	668 (98.4)	4 (0.7)	7 (5.7)	
Yes	11 (1.6)	553 (99.3)	115 (94.3)	
Cadre of healthcare sector				0.435^*^
Doctor	109 (16.10)	83 (14.9)	26 (21.3)	
Nurse	191 (28.1)	156 (28.0)	35 (28.7)	
Other health staff	72 (10.6)	59 (10.6)	13 (10.7)	
Student	186 (27.4)	156 (28.0)	30 (24.6)	
Support staff	121 (17.8)	103 (18.5)	18 (14.8)	

**Figure 2:**
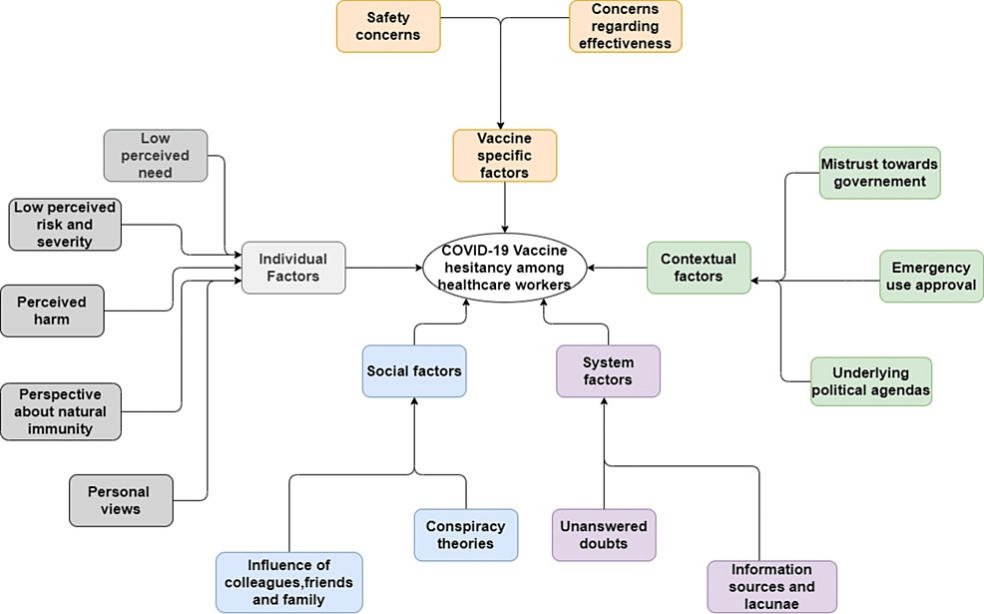
Thematic framework of COVID-19 vaccine hesitancy among healthcare workers

The consolidated description and explanation of phenomenon on thematic (vertical) and case-study mode (horizontal) is depicted in framework of Table 2. It is further supplemented by underlying philosophical positions and cognitive, emotional, and behavioral psychological perspectives. The framework matrix in essence shows the emergence of five themes namely individual, vaccine-related, social, system, and contextual factors. The cause-effect emergence of themes is further consolidated in Figure 2.

**Table 2:** The framework matrix

Participants	Thematic Premises					Philosophical Premises	Psychological Premises
	Individual factors	Vaccine-related factors	Social factors	System factors	Contextual factors		
25 years/M/Doctor	..not having any underlying medical conditions so if I get infected won’t be much problem		..rumors that this was decided due to lesser availability of vaccines.			Epistemological/methodological skepticism	Perceived barriers, lack conviction
33 years/M/Doctor	..worked in ward and ICU ..now I don’t think I need to get vaccinated	..made on emergency basis…all four stages couldn’t be completed			Government shifted its policies so early	Epistemological/methodological skepticism	Inability to draw a purpose, lack conviction
22 years/F/Student	For a healthy person, there is not much of a difference if they get vaccinated or not	.. are effective only for a short period of time unlike other vaccines	Parents were worried about me getting Vaccine A and told me to take Vaccine B	If more information were available, I would have taken it much before		Methodological skepticism	Anxious wrong choice, or lack conviction
28 years/M/Nurse		..do not know what the vaccine is made of, may change our DNA	Science is so advanced in Western countries, can modify our genes.	I read on Facebook and WhatsApp and found it not suitable	Fast approval of vaccine could also be because of the upcoming elections	Pyrrhonian/epistemological skepticism	Fear, anxiety, doubt, or insecurity
26 years/M/Nurse	Immunity acquired naturally of the disease is always better than the immunity acquired by vaccination	Maybe a vaccination for population control. Infertility in our children			Government is forcing us by saying this is mandatory	Epistemological/Pyrrhonian skepticism	Perceived barriers, ambiguous or conflicting thoughts
31 years/F/Doctor	..already been exposed to COVID while working in the ward, why vaccination?			Need for more and more data should be addressed	Vaccine has been developed in such a short time and approved for use	Methodological skepticism	Temperamental traits, past experiences, lack conviction
23 years/M/Student	My perspective is natural immunity is better than vaccination	Vaccines will reduce morbidity and mortality but I don't think that much reduction in infectivity is attained		I was having paracetamol allergy that I have a doubt whether this vaccine is recommended for this type of person	FDA provided early approval to these vaccines maybe to give popularity and support to the ruling party	Epistemological /methodological skepticism	Covet to avoid discomfort, lack conviction
40 years/M/Doctor	I think 3 days of fever after vaccination will affect my routine life activities			Since I had ended tuberculosis treatment 5 months before, I was not sure	Government should have had clear-cut guidelines earlier, then people would have been more confident	Pyrrhonian/methodological skepticism	Perceived barriers or obstacles, lack of motivation, covet to avoid discomfort
45 years/M/Paramedical staff	If in future a variant comes with severe complications, I might get vaccinated then	..some discrepancy, there are no antibodies in body even after taking 2 doses	..incident of side effect read in the news and everyone would panic		..have doubt that it has been developed quickly, research is lacking	Pyrrhonian/methodological/epistemological skepticism	Perceived barriers
25 years /F/ Nurse	Covid-19 is more or less like a Flu	We don't yet know about the side effects or long-term effects	My colleague had a severe adverse reaction which gave me concern		We still do not know how much reality the news channels and governments are saying	Moderate skepticism	Fear, anxiety, doubt, or insecurity
30 years/M/Doctor	I was procrastinating after hearing that we also need to take boosters every 6 months	Side effects, acute as well as long term were not fully evaluated				Pyrrhonian skepticism	Procrastination and refrainment
19 years/M/Student				I am from a rural area and there was no proper vaccination schedule there	For anything and everything we require vaccination certificate	Moderate skepticism	Social norms, peer pressure, and cultural factors
28 years/M/Support staff	COVID has been there for the last 2 years and nothing serious has happened		Seniors were convincing me while they themselves were not taking	During the initial phases if I had gotten a slot, but at that time the availability was very low.		Epistemological/moderate skepticism	Lack of motivation or a covet to avoid discomfort
23 years/F/Student	It’s a medication, and we don’t have any drugs without any side effects	I don’t find it harmful as such, but neither am I sure about its effectiveness			Compared to other vaccines available worldwide, I am not sure about effectiveness	Epistemological/methodological skepticism	Procrastination and refrainment, ambiguous or conflicting thoughts

**Figure 3:**
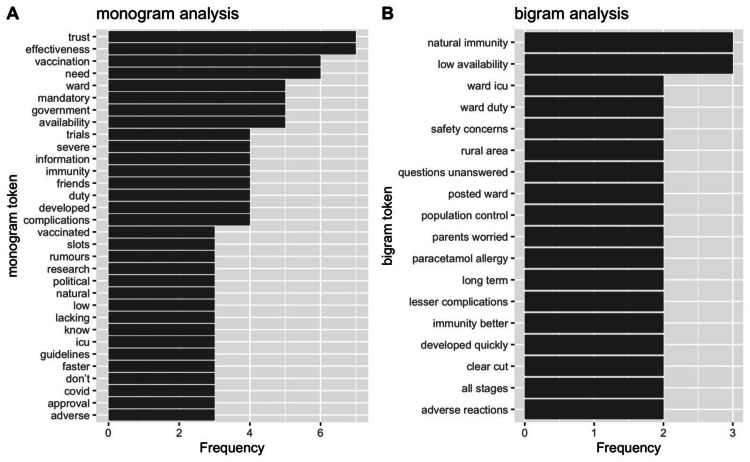
Bar graph showing the frequency distribution of monogram and bigram tokens

The semiquantitative approach using mono/bigrams decodes this further. Figure 3 shows the monogram and bigram analysis of the excerpt of the HCWs.

The most frequently used expressions in monogram were directed to “trust” and “effectiveness” of the vaccine and were closely followed by tokens of “need”, “mandatory”, and its “availability”. These tokens substantiate the individualistic aspirations to verify the strength of claims and the validity of evidence. This desire to get vindicated on the skeptical position by self and by others may be represented by next frequent words group like “information”, “friends”, “complications” etc. The other frequently used words like “rumors”, “slots”, “political”, “guidelines” give contextual aspect toward the hesitancy. Another set of word tokens such as “lacking”, “know”, “faster”, “approval” may suggest a desire to add conviction for improved and informed decision-making.

Similarly in bigram analysis “natural immunity” and “low availability” bigrams may indicate the quest to search for alternatives in the absence of certainty other bigrams like “population control”, “adverse reaction”, “safety concerns” sensed conspiracy theories that propagated, created a stigma for the vaccine shots.

**Figure 4:**
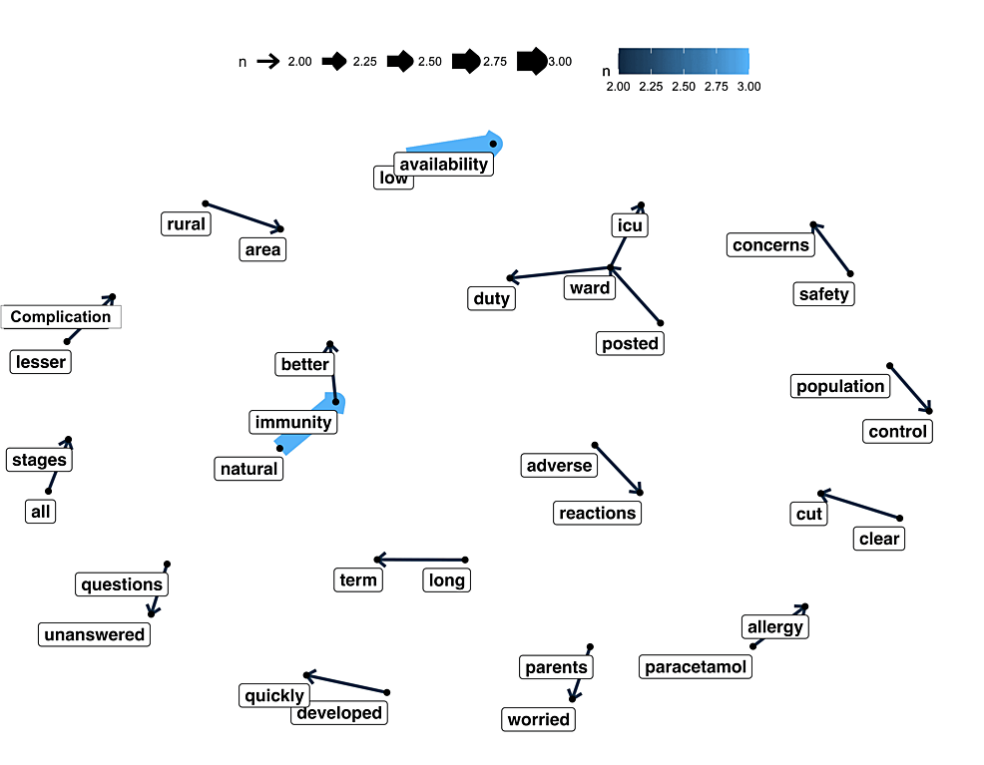
Network diagram illustrating correlation between words

Figure 4 shows the network analysis of the excerpts. As high solidity of the arrow depicts an increased frequency and assertion of the expression in the direction. For example, the central node being “natural” which is strongly linked to “immunity” is further linked to “better” inferring that natural immunity was considered as the preferred way of getting immunity. Similarly, the node “low” is strongly edged with “availability” indicating the availability issues of the vaccine. Another central node emerged was “ward” which had three edges to “duty”, “ICU” and “posted”, indicating the perception of acquiring immunity during duties in wards and ICU. Other bigrams like “long term”, “clear out”, “all stages” though have frequent appearances may not always give any clear information on hesitancy, therefore may not have strong edges and interconnections. The nodes may not have interconnectivity because the responses among the participants have been expressed in a varied way and eventually not clustered.

## Discussion

The COVID-19 vaccination campaign was the world’s largest vaccination drive and it was very successful in terms of coverage, management, innovative approaches, and risk protection [10,11].

With the unprecedented and insistent background of pandemic, it was a heroic effort taken by the government that had a longer and larger impact on India’s place in world geopolitics and the new world order [12]. This post-facto reality was blurred at those instances and societies all over the world were skeptical of COVID-19 vaccine safety and they had their own subjective assessments of risk perception and vaccine characteristics [13-15]. These internal struggles and conflicts may stem from fear, doubt, moral ambiguity, or conflicting desires on an individual plane. Larger and more organized systemic factors like sociocultural beliefs, system concerns, and complacency may also assign shape to it. Furthermore, concerns grew exponentially during the pandemic despite effective strategies for mass vaccination and intervention models to ameliorate vaccine literacy [16]. The hesitancy can create tension and suspense in high-stakes situations like COVID-19.

In our study,18% of HCWs demonstrated hesitancy aligned with the overall hesitancy reported in Madhya Pradesh and in other studies [17]. The advent of COVID-19 vaccines generated debates among professionals in the healthcare field. Their previous exposure to information and its translation into subjective knowledge might lead to an established mindset of having a reserved and conformist approach to vaccination [18,19]. Due to the inherent urgency of the development of new vaccines in a short period of time, approvals were expedited and development processes were shortened and improvised (REF). These improvisations created internal conflicts and concerns for inadequate evidence and expedited approval [20,21]. Several cross-sectional studies investigating COVID-19 vaccine hesitancy in HCWs have also reported widespread concerns about safety, efficacy, and potential side effects [21,22].

Scientific literacy and objectivism may not be translated into knowledge-behavior sequences, especially in ill-informed conditions. Propositions of perceived lower severity and threats from COVID-19 infection were also reported by other studies [23-25]. HCWs were very optimistic about their immunity and a hypothetical assumption of boosting immunity by serving in COVID-dedicated settings. This phenomenon may be decoded from the skepticize philosophical position where one may question the validity of the reasoning process itself (epistemological skepticism) as a limitation of absence of complete knowledge. Alternatively, it could be part of one’s belief in empirical verifications (methodological skepticism) and a tendency to challenge hypotheses. In the extreme cases of vaccine hesitancy, it could be a Pyrrhonian skepticism who advocates the state of suspension of judgment as a means to obtain freeness from assertive certainty. While those HCWs who received the vaccine in later days could be classified under moderate skepticism who eventually recognized the conditional nature of belief-system and the importance of acceptance. Overall skepticism contests the likelihood of accomplishing absolute certainty or complete knowledge about the world and encourages a cautious and skeptical attitude toward claims, theories, and dogmas. This skepticism was substantiated further by the dissemination of misinformation, influential opinion, and widespread disbeliefs about the vaccine [17,26,27]. Disbelief in vaccine is supplemented by the unusual beliefs that underlie the reluctance to receive vaccinations. One such misbelief of vaccine leading to infertility and the hidden motives behind mass vaccination strategy to bring about population control. Such notions existing within HCWs themselves shed light on the extent of misinformation and rumors that may be prevailing among the general population [17], and a reflection of symbolic motifs about broader societal or existential concerns.

Our findings suggest that vaccine hesitancy, even among well-informed individuals, hinges on a subjective evaluation of the risks and benefits involved, which can be swayed by incorrect and inadequate vaccine information.

Limitation

Our study uses a frequentist methodology while mining text or phrases. There seems to be a hidden threat of neglect of less frequent terms having deviant yet important aspect of vaccine hesitancy. This specific phenomenology of vaccine hesitancy in reference to COVID-19 may not fit routine vaccination altogether as the latter one is more matured, detailed, time-tested, and individualistic behavioral factors may dominate to systemic factors while this campaign was driven by pressing urgency and little was experienced like this before. Moreover, there may be systematic bias while comparing the experiences and concerns of HCWs with the general population as the former group is supposed to be closely associated and more informed on health behaviors and actions.

However, this study does contribute to the existing literature by providing valuable insights into the actual hesitancy levels among HCWs who had the opportunity to receive the vaccine for an extended period after a substantial duration of vaccine approval and availability. The methodological approaches adapted, the semiquantitative approach in our study complements qualitative insights, offering a more comprehensive understanding of the phenomenon of vaccine hesitancy.

## Conclusions

This study reveals a notable percentage of the HCW population had hesitancy toward the COVID-19 vaccine. Vaccine hesitancy by itself is a complex interplay of various determinants and can never be condensed to a single cause. Overall, hesitancy adds depth and nuance to narratives, reflecting the complexities of human nature and the dilemmas inherent in the human experience at intellectual and emotional levels. Through hesitant individuals, one may raise questions about free will, responsibility, and the consequences of indecision. Collectively our findings emphasize the necessity for healthcare organizations to promptly implement educational initiatives aimed at HCWs, targeted to address and alleviate their major concerns effectively.
